# An Integrated Machine Learning Scheme for Predicting Mammographic Anomalies in High-Risk Individuals Using Questionnaire-Based Predictors

**DOI:** 10.3390/ijerph19159756

**Published:** 2022-08-08

**Authors:** Cheuk-Kay Sun, Yun-Xuan Tang, Tzu-Chi Liu, Chi-Jie Lu

**Affiliations:** 1 Division of Hepatology and Gastroenterology, Department of Internal Medicine, Shin Kong Wu Ho-Su Memorial Hospital, Taipei 11101, Taiwan; 2 Graduate Institute of Business Administration, Fu Jen Catholic University, New Taipei City 24205, Taiwan; 3 School of Medicine, Fu Jen Catholic University, New Taipei City 24205, Taiwan; 4 School of Medicine, Taipei Medical University, Taipei 11031, Taiwan; 5 Department of Radiology, Shin Kong Wu Ho-Su Memorial Hospital, Taipei 11101, Taiwan; 6 Department of Medical Imaging and Radiological Technology, Yuanpei University of Medical Technology, Hsinchu 30015, Taiwan; 7 Artificial Intelligence Development Center, Fu Jen Catholic University, New Taipei City 24205, Taiwan; 8 Department of Information Management, Fu Jen Catholic University, New Taipei City 24205, Taiwan

**Keywords:** mammography, machine learning, breast cancer, national mammographic screening program, extreme gradient boosting

## Abstract

This study aimed to investigate the important predictors related to predicting positive mammographic findings based on questionnaire-based demographic and obstetric/gynecological parameters using the proposed integrated machine learning (ML) scheme. The scheme combines the benefits of two well-known ML algorithms, namely, least absolute shrinkage and selection operator (Lasso) logistic regression and extreme gradient boosting (XGB), to provide adequate prediction for mammographic anomalies in high-risk individuals and the identification of significant risk factors. We collected questionnaire data on 18 breast-cancer-related risk factors from women who participated in a national mammographic screening program between January 2017 and December 2020 at a single tertiary referral hospital to correlate with their mammographic findings. The acquired data were retrospectively analyzed using the proposed integrated ML scheme. Based on the data from 21,107 valid questionnaires, the results showed that the Lasso logistic regression models with variable combinations generated by XGB could provide more effective prediction results. The top five significant predictors for positive mammography results were younger age, breast self-examination, older age at first childbirth, nulliparity, and history of mammography within 2 years, suggesting a need for timely mammographic screening for women with these risk factors.

## 1. Introduction

Breast cancer is the most prevalent cancer in women worldwide and is also the leading cause of cancer-related deaths in women [1]. The results of a previous meta-analysis of randomized clinical trials between 2009 and 2015 demonstrated the beneficial role of screening for breast cancer and early detection in reducing breast cancer mortality among women aged 50–69 [2]. The 2015 American Cancer Society Guidelines recommend that women undergo regular mammography starting at the age of 45 and biennial screening mammography after age 55 and then continue screening even if they are in good health into an advanced age [3]. A report from the 2013 National Health Interview Survey of America showed that 69.1% of women aged 50 or older adhered to the breast cancer screening guidelines every 2 years, whereas mammography screening rates remained much lower in Asian countries [4].

In Taiwan, the government has promoted biennial mammograms for women aged 50–69 since 2004 and expanded the recommendation in 2009 to include women aged 45–69. The screening program was further extended in 2010 to include high-risk women aged 40–44 [5]. All participants must complete a risk factor questionnaire before the mammographic examination. The reported risk factors for breast cancer are modifiable and non-modifiable. Although factors associated with lifestyle (e.g., alcohol consumption, being overweight or obese, parity, physical inactivity, and using certain medications, e.g., oral contraceptives) [6,7] are modifiable, other factors such as age, sex, genetic characteristics (including ethnicity and family or personal history of breast cancer), and early menarche or menopause are non-modifiable [8]. However, the relative importance of the reported risk factors remains controversial [9].

Despite improvements in health delivery systems, barriers to adherence to mammography screening may also include personal factors such as age, lack of knowledge of the risk factors, lower economic status, and underlying chronic disease [5,8,10]. Therefore, identifying the risk factors associated with mammographic anomalies is critical for targeting at-risk individuals to effectively allocate diagnostic and therapeutic resources. Machine learning (ML) is a branch of artificial intelligence that develops and utilizes algorithms for the data to mimic the way that humans learn [11,12]. ML techniques have been widely and successfully used in healthcare and medical research [11,12,13,14,15]. However, to the best knowledge of the authors, no studies have investigated the important risk factors related to predicting positive mammographic findings based on questionnaire-based demographic and obstetric/gynecological parameters using ML algorithms. Different ML algorithms may generate different information when analyzing the data based on their different mechanisms. Integrating the benefits of different ML techniques could provide more useful information to help and support decision making [16]. Thus, we aim to propose an integrated ML predictive scheme to effectively produce mammographic anomaly prediction results for high-risk individuals and recognize the significant risk factors by incorporating the information acquired from the risk factor questionnaires and mammographic findings. 

The proposed integrated ML scheme combines the benefits of two well-known ML algorithms, namely, least absolute shrinkage and selection operator (Lasso) logistic regression and extreme gradient boosting (XGB), to generate complete and adequate model prediction and important risk factor identification results. XGB and Lasso methods are both widely and successfully used techniques in breast cancer/mammography researches [17,18,19,20]. They are also commonly used approaches to selecting the critical predictor variables in healthcare and medical informatics applications [21,22]. XGB could effectively identify important variables when the variables have nonlinear and/or high dimensionality interactions [23,24]. However, it cannot provide the direction of influence of the selected important variables. Alternatively, Lasso can present the influence directions (positive or negative) of the selected predictor variables on the target variable. However, it may not adequately select significant variables when the variables contain multivariate and nonlinearly interacting information. In the proposed scheme, the XGB model was first used to select and rank the important predictor variables. The ranked variables were then used to generate stepwise-based variable ranking combinations. The Lasso method was used for the combinations to generate the final predictive models.

## 2. Methods

### 2.1. Study Design and Protocol

This study retrospectively reviewed the risk factor questionnaires completed by women who participated in a national mammographic screening program at a single tertiary referral hospital in northern Taiwan (i.e., Shin-Kong Wu Ho-Su Memorial Hospital, Taipei) between January 2017 and December 2020. The questionnaire and survey process were uniformly designed and set by the Ministry of Health and Welfare in Taiwan. We retrieved information from the medical records of the participants. This information includes age, age at menarche, body height, body mass index (BMI), education level, history of significant diseases, experience of breast self-examination and mammography within the preceding 2 years, history of breast surgery, age at first childbirth, parity, breastfeeding, reproductive lifespan, age at initiation and duration of hormone replacement therapy, age at initiation and duration of oral contraceptive use, and the number of relatives with confirmed breast cancer. Mammograms of the participants were also recorded.

All women who underwent the national mammography screening program and completed a risk factor questionnaire during the study were eligible for the current study. Exclusion criteria were (1) participants with body weight below 35 kg or over 120 kg, (2) questionnaires with illogical data (e.g., contradictory reporting of age at menarche and first childbirth) or incomplete information, and (3) history of breast cancer. The study protocol and procedure were reviewed and approved by the Research Ethics Review Committee of Shin Kong Wu Ho-Su Memorial Hospital, which also waived the requirement for informed consent from the participants before the routine examination procedure (No. 20210703R). 

**Figure 1:**
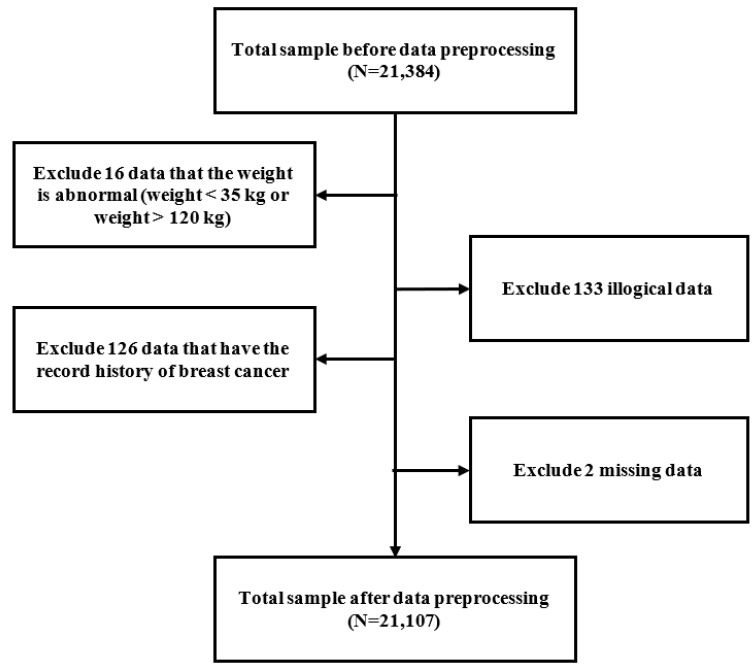
Data preprocessing flow chart.

Out of a total of 21,384 questionnaires collected from the participants before their mammographic screening during the study period, those completed by 16 women with a body weight below 35 kg or over 120 kg, 133 questionnaires with illogical data, and 2 questionnaires with missing data were excluded. Finally, we used data from 21,107 questionnaires to analyze in the current study (Figure 1).

### 2.2. Study Parameters and Definitions

To investigate the associations between participants’ characteristics and mammographic findings, 18 patient variables were assigned as the predictor variables. These are X1: age, X2: body height, X3: age at menarche, X4: body mass index (BMI), X5: education level, X6: history of major diseases, X7: breast self-examination, X8: mammography within the preceding 2 years, X9: history of breast surgery, X10: age at first childbirth, X11: parity, X12: breastfeeding, X13: reproductive lifespan, X14: age at starting hormone replacement therapy, X15: duration of hormone replacement therapy, X16: age at starting oral contraceptive therapy, X17: duration of oral contraceptive use, and X18: number of relatives with confirmed breast cancer. 

The mammographic findings (Y), categorized as either negative or positive based on the need for this study, were used as the target variables. The former were defined as mammograms without anomalies and those showing benign lesions (i.e., The Breast Imaging Reporting and Data System (BI-RADS) categories 1 and 2, respectively). The latter referred to “need additional imaging or prior examinations” (BIRADS 0), “probably benign” (BIRADS 3), “suspicious” (BIRADS 4), “highly suggestive of malignancy” (BIRADS 5), and “known biopsy-proven” (BI-RADS 6).

For the current study, the education level (X5) was divided into five categories: primary school, lower secondary school, upper secondary school, university, and postgraduate. The history of major diseases (X6) was divided into three categories: without major diseases, with benign comorbidity, and cancer other than breast cancer. There were three categories of breast self-examination (X7): negative self-examination results; never performed self-examination; and detected mass, pain, or tenderness during self-examination. There were four categories of age at first childbirth (X10): below 21, between 21 and 34, greater than or equal to 35, and did not experience childbirth. Parity (X11) was divided into five categories from never to more than or equal to four times. Breastfeeding (X12) was divided into three categories: nulliparous, not breastfeeding, and breastfeeding. Reproductive lifespan (X13), the time from the onset of menarche to the date of menopause or the date of examination for those who were premenopausal, was divided into five categories: below or equal to 24, between 25 and 29, between 30 and 34, between 35 and 39, and equal to or over 40 years. There were six categories regarding the age of starting hormone replacement therapy (X14): less than 30, between 30 and 39, between 40 and 49, between 50 and 59, greater than or equal to 60, and those who did not receive hormone replacement therapy. For the duration of hormone replacement therapy (X15), there were three categories: those who did not receive hormone replacement therapy, those who underwent therapy for less than 5 years, and those being treated for more than or equal to 5 years. Focusing on the age at which oral contraceptives were started (X16), the three categories included less than or equal to 25, greater than 25, and never received oral contraceptives. The duration of oral contraceptive use (X17) comprised three categories: those who did not receive contraceptives, those being treated for less than or equal to 5 years, and those who underwent treatment for more than 5 years. The number of relatives with confirmed breast cancer (X18), which refers to the number of relatives who had breast cancer regardless of age, was classified into three categories: those who did not have a relative with breast cancer, those who had one relative, and those who had more than or equal to two relatives with confirmed breast cancer. A binary outcome (yes or no) was assigned to the parameters of mammography within the preceding two years (X8) and a history of breast surgery (X9).

Table 1 presents the statistics of the 18 predictor variables considered risk factors for mammographic anomalies (Y) from the questionnaires before the mammography screening. The mean age (X1) of participants was 55.18 ± 7.13 years. Their educational levels were mainly upper secondary school and university (35% and 33%, respectively). Sixty percent of all participants underwent mammography within the preceding 2 years. All participants were classified into three categories according to breast self-examination (X7): those with negative self-examination results (73%), those who never performed self-examination (22%), and those who detected a mass or experienced pain or tenderness during self-examination (5%). Most participants had their first childbirth between the ages of 21 and 34 (75%). The percentages of individuals who did and did not breastfeed were similar (42% and 45%, respectively). Parity was divided into five categories (X11) from zero to more than or equal to four times. Most of them had two full-term pregnancies (44%).

**Figure 2:**
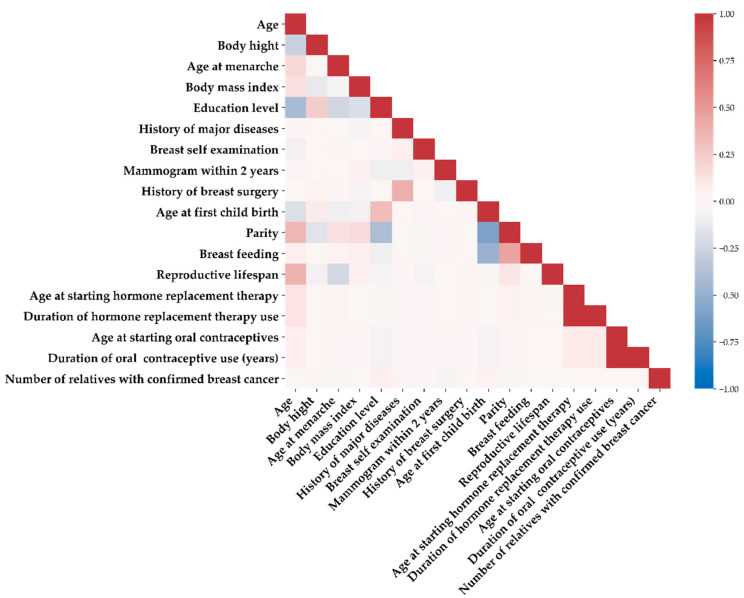
Heatmap visualization of the correlation matrix between the 18 variables.

Figure 2 shows a heatmap visualization of the correlation matrix between the 18 predictor variables to explore the relationship between the variables using Spearman’s correlation coefficient [25]. As shown in the figure, the higher the correlation between the variables, the darker the color. The colors red and blue indicate positive and negative correlations, respectively. According to the correlation matrix, the correlations among the 18 variables were compatible with the clinical presentations. The three most remarkable correlations were as follows: First, early age at first childbirth was associated with an increased number of pregnancies. This association was consistent with that reported in a previous study [26]. Second, a younger age of starting hormone replacement therapy correlated to a longer duration of hormonal treatment. Third, a younger age of beginning oral contraceptives was positively related to the duration of contraceptive use.

### 2.3. Proposed Integrated ML Scheme

This study proposes an integrated ML scheme that utilizes the advantages of Lasso and XGB methods to construct predictive models and identify important predictor variables for predicting mammographic anomalies in high-risk individuals. 

XGB is implemented under the gradient-boosting framework, designed to be highly efficient, flexible, and portable. It belongs to a broader collection of tools under the umbrella of the distributed ML community and is a research project started by Chen [27]. Logistic regression is a widely used classic regression algorithm [19,28] that focuses on binary classification problems by calculating the natural logarithm of the odds ratio (logit) [29]. To obtain a more accurate prediction using shrinkage, Lasso is a regularization technique used over the logistic regression method, where data values are shrunk toward a central point as the mean [30].

**Figure 3:**
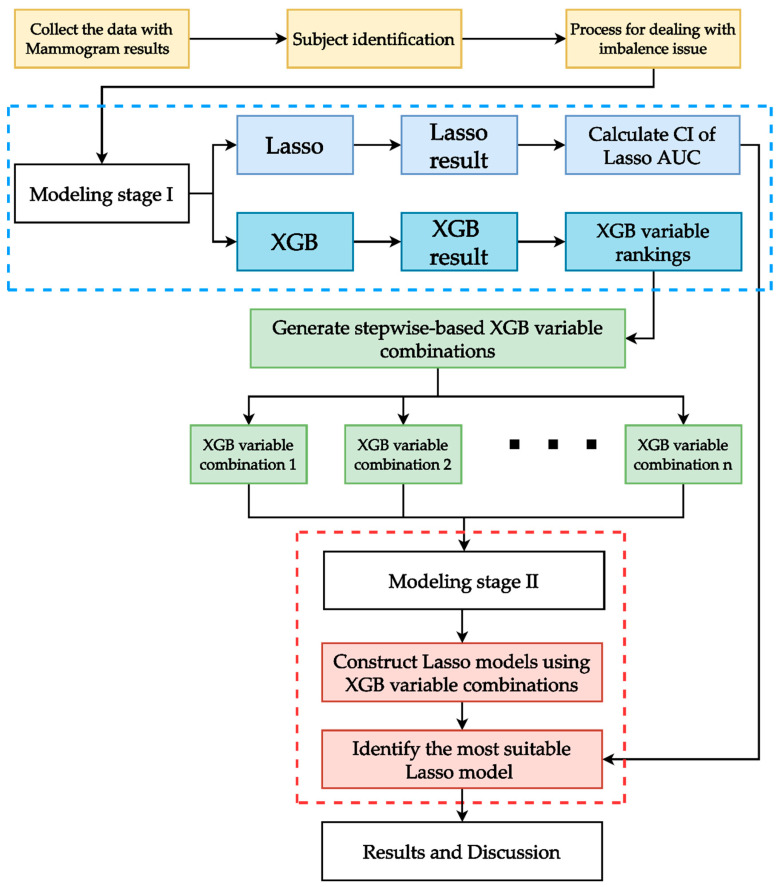
Proposed integrated ML scheme.

Figure 3 shows the flowchart of the proposed integrated ML scheme. As shown in the figure, after collecting the data with the mammogram results, a series of data preprocessing processes (e.g., missing value elimination, outlier detection, and categorical coding labels) were utilized to clean the data. The data quality was checked to ensure that the survey had been correctly completed; for example, samples with illogical time recordings were excluded.

The classes of the target variable (i.e., mammogram findings) were skewed, and most data contained negative cases. Training the model with imbalanced data may cause the model to spend the most time learning the most negative cases and miss a large amount of information provided by the positive cases. This study utilized a bootstrap-based undersampling technique to address the imbalance issues. The main concept of the undersampling technique is to balance the data by decreasing the amount of the majority class (negative cases in this study) until the achievement of the desirable proportion between both classes [31]. The equal proportion of majority and minority is used [32]. The bootstrapping method is a well-known sampling strategy, which is sampling with replacement to decrease the amount of the majority class [33].

There are two stages in the modeling of the proposed scheme. In modeling stage I, Lasso logistic regression was fitted with all variables (called the full model) to calculate the confidence interval (CI) of the performance index area under the receiver operating characteristic (ROC) curve (AUC) for the method. Calculating the CI of the AUC can indicate the stability of the model’s performance. Simultaneously, XGB was also fitted with all variables in this stage to obtain the variable importance rankings, which were used to generate the variable combinations. 

It is important to remember that ML methods can highlight multivariate interaction effect information. Thus, the variable rankings obtained from the ML method in stage I may provide more information. Furthermore, using stepwise algorithms to generate stepwise-based ranked variable combinations (SRVCs) to model different predictive models makes it easier to evaluate the impact of the different SRVCs on the AUC values of the models. SRVCs consist of the top-ranking variable as the first combination and the top-two ranking variables as the second combination. To generate SRVCs based on XGB in stage I, the variable importance rankings obtained from XGB generate XGB-based SRVCs (called XGB-SRVCs). 

In the stage II modeling of the proposed scheme, to obtain adequate prediction and beneficial risk factor identification results, Lasso logistic regression was used for XGB-SRVCs to generate different predictive models. The AUC of each combination of XGB-SRVCs was compared to the CI of the AUC of the Lasso logistic full model. Among all XGB-SRVCs, the most suitable variable combination was identified based on the lower bound of the CI of the AUC of the Lasso logistic full model, that is, among all variable combinations that exceed the lower CI of the AUC of the Lasso logistic full model, the combination that contains the fewest variables is the most suitable. Finally, a logistic model was developed with the selected suitable variable combination of XGB-SRVCs to reveal the effect direction of important predictor variables.

The experiment was implemented in Python version 3.8.8 (Guido van Rossum: The Hague, The Netherlands) [34] and Jupyter Notebook version 6.3.0 (Fernando Pérez: Medellin, Colombia) [35]. A bootstrap-based undersampling technique was implemented with the imbalanced-learn package version 0.8.0 (Guillaume Lemaître: Palaiseau, France) [36]. For every modeling process, the data were randomly split into 80% for training and 20% for testing. Note that the balancing technique is only utilized in the training stage when modeling. Further, fivefold cross-validation was utilized for the hyper-parameter tuning. All the modeling processes for the Lasso and XGB were repeated one hundred times. Lasso was constructed with the Scikit-learn package API with version 0.24.2 (Fabian Pedregosa: Paris, France) [37,38], XGB was constructed with the xgboost package with version 1.3.3 (Tianqi Chen: Pittsburgh, PA, USA) [27], and cross-validation and hyper-parameter tuning were constructed with the Scikit-learn API [37,38]. Because the data for this study are a binary classification, the primary metric used to evaluate the performance of the model is the AUC. This study also provided sensitivity, specificity, and accuracy.

## 3. Results

### 3.1. Models with and without Data Balancing

Table 2 presents the model results of the Lasso logistic regression and XGB with and without the balancing techniques for comparison. Note that the oversampling technique is also used for comparison as it is one of the most common methods of dealing with class imbalance issues [31,39]. As presented in the table, the AUC of both methods was close with and without the balancing techniques. However, there were significant differences in sensitivity without the balancing techniques, that is, the sensitivity and specificity of the Lasso method were 0 and 100, whereas those of the XGB were 3.08 and 98.35. The low sensitivity and specificity indicated that the model predicted negative cases well but was less effective for the positive cases.

The model results can be improved by using the balancing techniques before utilizing Lasso and XGB methods. The table shows that the sensitivity and specificity of the Lasso and XGB methods with undersampling were 60.97, 58.64, 63.00, and 52.87, respectively, whereas those with oversampling were 60.67, 58.89, 87.65 and 15.26, respectively. Table 2 also shows that the Lasso and XBG methods with undersampling can provide better AUC and more stable sensitivity and specificity results. Therefore, the CI of the AUC of the Lasso full model (CI [62.59, 63.01]) was calculated based on the AUC results of the Lasso method with undersampling. The variable importance rankings of the Lasso and XGB models with undersampling are presented in Table 3.

### 3.2. Variable Importance Ranking

**Figure 4:**
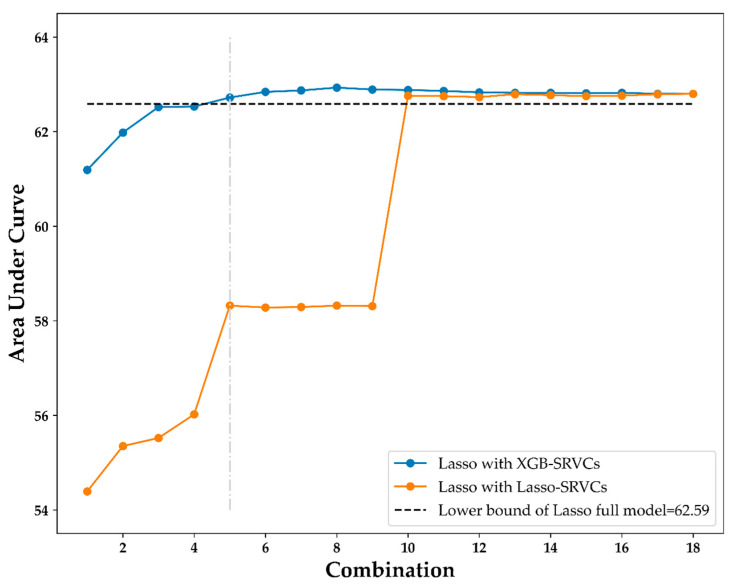
Lasso results comparison using XGB-SRVCs and Lasso-SRVCs.

Table 3 shows the variable importance rankings of the XGB and Lasso models. These models generate different variable importance rankings, although they have similar model performances. As presented in the table, for instance, age is ranked one by XGB, but ranked ten by Lasso, and breast self-examination is ranked two by XGB and ranked one by Lasso. The XGB variable importance ranking generates the XGB-SRVCs and is then used in the Lasso logistic regression method to generate the different models. Table 4 and Figure 4 present the results.

### 3.3. Lasso Results with XGB-SRVCs

Table 4 shows the first five combinations (C1–C5) of the XGB-SRVCs and the AUC of the Lasso method using each variable combination as a predictor variable. As presented in the table, although the number of variables increased, the AUC also increased and approached the lower bound of the CI of the AUC of the Lasso full model (62.59). For the fifth combination, the AUC was 62.72, which exceeded the full model’s AUC lower bound (62.59). These results indicate that the effectiveness of these five variables in training the Lasso model is the same as that of all 18 variables. Therefore, the number of variables required when training the Lasso model is reduced.

Meanwhile, using the same concept as the XGB-SRVCs, the Lasso-SRVCs are also generated according to the variable importance rankings of Lasso (Table 3). Figure 4 shows the results of the Lasso model using various combinations of XGB-SRVCs and Lasso-SRVCs as predictor variables. The black horizontal dashed line in Figure 4 indicates the CI lower bound of the AUC of the Lasso full model (62.59). As also shown in the figure, the Lasso model with the XGB-SRVCs reaches the CI lower bound using the fifth combination (C5), indicated by the vertical light-grey dot and dashed line. However, using the fifth combination, the Lasso model with the Lasso-SRVCs did not reach the CI lower bound until the tenth combination. Figure 4 shows that the Lasso model using the XGB-SRVCs was more efficient than using the Lasso-SRVCs, that is, the model became stable when the AUC reached the lower bound of the CI. The top five important variables ranked by XGB, namely, age, breast self-examination, age at first childbirth, parity, and mammography within 2 years, can be regarded as the most suitable variables for Lasso modeling.

The Lasso model based on the five most important variables can generate the following logistic equation which can be used to predict the mammographic anomalies of a high-risk individual:

Mammogram findings=2.264–0.046×Age+0.242×Breast self−exam+0.103×Age of first childbirth–0.031×Parity+0.107×Mammography within two years.

**Figure 5:**
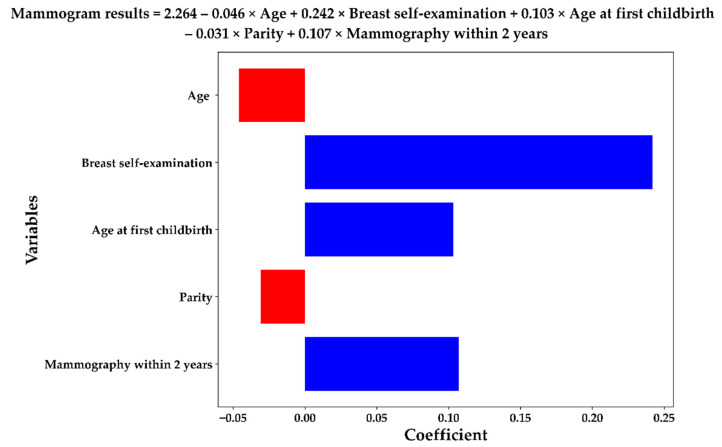
Lasso equation with the selected five important variables.

Figure 5 presents this logistic equation in a bar chart and the equation is presented at the top of the figure. The positive and negative coefficients of the variables in the equation are marked in blue and red, respectively. As shown in the figure, age and parity were negatively correlated with the mammogram findings. According to the equation, younger age; the presence of a mass, pain, or tenderness in breast self-examination; older age at first childbirth; decreasing parity; and mammography within the preceding 2 years were the major factors predicting positive mammographic outcomes. The proposed scheme identified five important variables, two of which negatively correlated with positive mammographic outcomes.

## 4. Discussion

Despite the popularity of mammography as a screening tool for breast cancer, no reports have focused on the risk factors associated with mammographic findings. In contrast to most previous studies that focused on the identification of the risks factors associated with established breast cancers, the current study investigated the risk factors significantly related to positive mammographic findings based on a simple questionnaire so that an increased level of attention can be paid to the high-risk population to improve the detection rate of breast cancer as well as allow early advanced diagnostic studies and treatments. In addition, two mixed machine learning approaches (XGB-SRVCs and Lasso-SRVCs) were compared to identify the best method that could more effectively predict positive mammographic findings. This is the first study that addresses this issue by incorporating demographic and clinical information into mammographic findings through an ML scheme. This study used information from questionnaires and ML analysis to assess the associations between 18 factors and mammographic screening results. Our findings showed that XGB feature rankings were more efficient than those of Lasso. Meanwhile, age, breast self-examination, age at first childbirth, parity, and mammography within 2 years were the top five significant variables predicting a positive result for mammographic screening for breast cancer. Therefore, our results may have significant implications for clinical practice.

Our results showed that age was the most crucial predictor of mammographic findings, as reflected by the negative correlation between age and positive mammographic findings. Compared with the United States, a previous investigation reported that breast cancer in Taiwan is diagnosed at a younger age, with a higher and increasing incidence [5]. However, because younger women are more likely to have dense breasts than older women, screening in the younger population may be associated with an increased probability of false-positive results. Breast self-examination (X7) was our study’s second most common variable. Few randomized trials have focused on the efficacy of breast self-examination in detecting breast cancer [40]. One study in China, which randomly divided 266,064 women into a breast self-examination instruction group and a control group, demonstrated that the total number of breast lesions required to perform biopsies was remarkably higher in the instruction group than that in the control group [41]. Therefore, the results of that study support our findings of an increased likelihood of a positive mammogram among those who underwent breast self-examination. 

Age at first childbirth (X10) was the third most important variable for predicting positive mammographic results in this study. Our findings demonstrated that early age at first childbirth was associated with a reduced risk of positive mammography findings. Previous studies have consistently shown a protective effect of first full-term birth at an early age against the development of breast cancer [42], contributing to pregnancy-induced physiological changes in mammary tissue that may render breast cells less vulnerable to neoplastic transformation [43]. 

The fourth most important risk factor is parity (X11). Previous studies have shown that nulliparous women are at a higher risk of breast cancer than parous women, with the increased estimated relative risk of the former ranging from 1.2 to 1.7 compared with the latter [44]. In contrast, although parous women have been reported to have an increased risk of developing breast cancer within the first few years of delivery relative to their nulliparous counterparts, parity is believed to confer a protective effect for decades after delivery [45]. A body of evidence has consistently demonstrated a reduced risk with increasing pregnancies [44,46,47].

The fifth risk factor in the present study was the participants receiving mammography within the preceding 2 years (X8). This finding may be partially attributed to a higher probability of pre-existing lesions or abnormal mammographic results requiring follow-up examinations in this population than in those without previous breast anomalies. In addition, individuals with higher screening adherence, such as those with a higher education level, have exhibited an increased incidence of breast cancer, but a decreased mortality [48,49].

Although various risk factors for breast cancer among Asian women have previously been reported, including early menarche, late menopause, family history of breast cancer, high body mass index, being overweight, and obesity [8], this study is the first to investigate the predictive values and ranking of the factors related to positive mammographic findings using information from the breast cancer risk factor questionnaire and two artificial intelligence methods. In this study, ML analyzed 18 variables associated with positive mammography screening results. A comparison between the Lasso model with XGB-SRVCs and Lasso model with Lasso-SRVCs demonstrated that the former not only required few variables but also had a higher AUC in predicting positive mammography results. Further studies are warranted to elucidate the importance of “age”, which was the most important predictive variable in our study, regarding the best cutoff point for maximizing its predictive value. Moreover, collecting more cohort data and/or useful predictors from medical records for model building may improve the performance of the proposed framework. It could be another important future research direction.

## 5. Conclusions

By analyzing 18 factors possibly associated with the risk of positive mammography screening for breast cancer using the proposed integrated ML scheme, we identified younger age, breast self-examination, older age at first childbirth, nulliparity, and history of mammography within the preceding 2 years as the top five significant variables for predicting positive mammography results. Our findings suggest a benefit for women with the identified risk factors for timely mammographic screening.

## Figures and Tables

**Table 1:** Demographic and clinical characteristics of participants.

Characteristics	Metrics
	**Mean (SD)**
**X1: Age**	55.16 (7.13)
**X2: Height**	157.56 (5.35)
**X3: Age at menarche**	13.87 (1.61)
**X4: Body mass index**	23.65 (3.57)
	**N (%)**
**X5: Education level**	
0: Primary school	3036 (14%)
1: Lower secondary school	2668 (13%)
2: Upper secondary school	7295 (35%)
3: University	6891 (33%)
4: Postgraduate	1217 (6%)
**X6: History of major diseases**	
0: No	17,809 (84%)
1: Benign	2702 (13%)
2: Cancer (other than breast)	596 (3%)
**X7: Breast self-examination**	
0: Breast self-exam negative	15,435 (73%)
1: Never breast self-exam	4540 (22%)
2: Mass or pain or tenderness	1132 (5%)
**X8: Mammography within 2 years**	
0: No	8338 (40%)
1: Yes	12,769 (60%)
**X9: History of breast surgery**	
0: No	19,345 (91%)
1: Yes	1762 (9%)
**X10: Age at first childbirth**	
0: Age < 21	1635 (8%)
1: 21 ≤ Age < 35	15,741 (75%)
2: Age ≥ 35	1037 (5%)
3: No childbirth	2694 (13%)
**X11: Parity**	
0: 0 times	2694 (13%)
1: 1 time	3136 (15%)
2: 2 times	9319 (44%)
3: 3 times	4688 (22%)
4: ≥4 times	1270 (6%)
**X12: Breastfeeding**	
0: Nulliparous	2694 (13%)
1: No	9554 (45%)
2: Yes	8859 (42%)
**X13: Reproductive lifespan**	
0: lifespan < 25	430 (2%)
1: 25 ≤ lifespan < 30	1272 (6%)
2: 30 ≤ lifespan < 35	6357 (30%)
3: 35 ≤ lifespan < 40	9447 (45%)
4: lifespan ≥ 40	3601 (17%)
**X14: Age at starting hormone replacement therapy**	
0: No use	19,655 (93%)
1: Age ≥ 60	50 (<1%)
2: 50 ≤ Age < 60	681 (3%)
3: 40 ≤ Age < 50	590 (3%)
4: 30 ≤ Age < 40	103 (<1%)
5: Age < 30	28 (<1%)
**X15: Duration of hormone replacement therapy use**	
0: duration = 0	19,655 (93%)
1: 0 < duration < 5	1027 (5%)
2: duration ≥ 5	425 (2%)
**X16: Age at starting oral contraceptives**	N%
0: No use	19,903 (94%)
1: Age > 25	754 (4%)
2: Age ≤ 25	450 (2%)
**X17: Duration of oral contraceptive use (years)**	
0: No use	19,903 (94%)
1: year ≤ 5	929 (4%)
2: year > 5	275 (1%)
**X18: Number of relatives with confirmed breast cancer**	
0: 0	18,746 (89%)
1: 1	2190 (10%)
2: ≥2	171 (1%)
**Y: Mammogram findings**	
0: Negative	18,089 (86%)
1: Positive	3018 (14%)

**Table 2:** Comparison of models with and without the balancing technique.

Data Balancing	Methods	Metrics			
		Accuracy	Sensitivity	Specificity	AUC
		Mean (SD)	Mean (SD)	Mean (SD)	Mean (SD)
Without balancing	Lasso	85.70 (0.48)	0.00	100 (0.01)	63.00 (1.11)
	XGB	84.73 (3.17)	3.08 (8.91)	98.35 (5.12)	63.22 (1.04)
With undersampling balancing	Lasso	58.97 (0.94)	60.97 (2.03)	58.64 (1.19)	62.80 (1.05)
	XGB	54.30 (16.56)	63.00 (15.38)	52.87 (21.83)	62.32 (1.25)
With oversampling balancing	Lasso	58.23 (0.83)	60.67 (1.89)	58.89 (0.99)	62.66 (1.10)
	XGB	25.62 (6.69)	87.65 (6.72)	15.26 (8.86)	59.26 (1.19)

**Table 3:** Variable importance rankings of Lasso and XGB models.

Rank	Variable	Lasso Method	Variable	XGB Method
1	X7	Breast self-examination	X1	Age
2	X8	Mammography within 2 years	X7	Breast self-examination
3	X15	Duration of hormone replacement therapy	X10	Age at first childbirth
4	X6	Major diseases	X11	Parity
5	X10	Age at first childbirth	X8	Mammography within 2 years
6	X16	Age at starting oral contraceptives	X6	Major diseases
7	X14	Age at starting hormone replacement therapy	X5	Education level
8	X17	Duration of oral contraceptive use	X4	Body mass index
9	X9	History of breast surgery	X16	Age at starting oral contraceptives
10	X1	Age	X13	Reproductive lifespan
11	X12	Breastfeeding	X2	Body height
12	X18	Number of relatives with confirmed breast cancer	X14	Age at starting hormone replacement therapy
13	X5	Education level	X12	Breastfeeding
14	X11	Parity	X18	Number of relatives with confirmed breast cancer
15	X13	Reproductive lifespan	X3	Age at menarche
16	X3	Age at menarche	X9	History of breast surgery
17	X4	Body mass index	X17	Duration of oral contraceptive use
18	X2	Body height	X15	Duration of hormone replacement therapy

**Table 4:** Lasso result with XGB-SRVRCs.

XGB-SRVCs Combinations	Variables	AUC
C1	(Age)	61.19
C2	(Age) + (Breast self-examination)	61.98
C3	(Age) + (Breast self-examination) + (Age at first childbirth)	62.52
C4	(Age) + (Breast self-examination) + (Age at first childbirth) + (Parity)	62.53
C5	(Age) + (Breast self-examination) + (Age at first childbirth) + (Parity) + (Mammography within 2 years)	62.72

## Data Availability

Data are available on request due to privacy/ethical restrictions. The questionnaire can be requested from the Ministry of Health and Welfare in Taiwan.
